# Commentary: Quality nutrition care is integral to the Oncology Care Model

**DOI:** 10.1007/s00520-021-06436-x

**Published:** 2021-07-23

**Authors:** Mary Beth Arensberg, Beth Besecker, Laura Weldishofer, Susan Drawert

**Affiliations:** 1 Abbott Nutrition Division of Abbott, Columbus, OH USA; 2 US Oncology, Cincinnati, OH USA; 3 Abbott, Minneapolis, MN USA

**Keywords:** Oncology Care Model (OCM), Nutrition, Malnutrition, Nutrition-focused quality improvement programs (QIPs), Health outcomes

## Abstract

The Oncology Care Model (OCM) is a US Centers for Medicare & Medicaid Services (CMS) specialty model implemented in 2016, to provide higher quality, more highly coordinated oncology care at the same or lower costs. Under the OCM, oncology clinics enter into payment arrangements that include financial and performance accountability for patients receiving chemotherapy treatment. In addition, OCM clinics commit to providing enhanced services to Medicare beneficiaries, including care coordination, navigation, and following national treatment guidelines. Nutrition is a component of best-practice cancer care, yet it may not be addressed by OCM providers even though up to 80% of patients with cancer develop malnutrition and poor nutrition has a profound impact on cancer treatment and survivorship. Only about half of US ambulatory oncology settings screen for malnutrition, registered dietitian nutritionists (RDNs) are not routinely employed by oncology clinics, and the medical nutrition therapy they provide is often not reimbursed. Thus, adequate nutrition care in US oncology clinics remains a gap area. Some oncology clinics are addressing this gap through implementation of nutrition-focused quality improvement programs (QIPs) but many are not. What is needed is a change of perspective. This paper outlines how and why quality nutrition care is integral to the OCM and can benefit patient health and provider outcomes.

**Supplementary Information:**

The online version contains supplementary material available at 10.1007/s00520-021-06436-x.

## Introduction

As the global burden of cancer continues to rise, it is expected to become the leading barrier to increased life expectancy [1]. Simultaneously the costs of cancer care continue to surge and thus government payers such as the United States (US) Centers for Medicare & Medicaid Services (CMS) are seeking innovative solutions.

The Oncology Care Model (OCM) is a CMS specialty model implemented in 2016, to provide higher quality, more highly coordinated oncology care at the same or lower costs. Under the OCM, oncology clinics enter into payment arrangements that include financial and performance accountability for patients receiving chemotherapy treatment. In addition, they commit to providing enhanced services, including care coordination, navigation, and following national treatment guidelines [2].

Nutrition is a component of best-practice cancer care, yet it may not be addressed by OCM providers. This is surprising because poor nutrition has a profound impact on cancer treatment and survivorship [3]. What is needed is a change of perspective. This paper outlines how and why quality nutrition care is integral to the OCM.

## The persistent malnutrition problem

There is strong evidence associating poor nutrition status with decreased tolerance to chemotherapy and radiation treatment, increased lengths of hospital stay, lower quality of life, and mortality [4]. Poor nutrition in cancer most often manifests as protein-energy undernutrition/malnutrition and is a persistent problem; up to 80% of older patients with cancer develop malnutrition [5, 6] and patients’ nutritional issues frequently change over time and are complicated by cancer cachexia [7]. Guidelines and positions on the management of these conditions therefore emphasize early diagnosis of malnutrition and cachexia [8], the importance of dietary counseling [9], and supportive care including nutritional support [10]. However, only about half of US ambulatory oncology settings screen for malnutrition, registered dietitian nutritionists (RDNs) are not routinely employed by oncology clinics, and the medical nutrition therapy they provide is often not reimbursed [11]. Thus, adequate nutrition care in US oncology clinics remains a gap area [3].

Some oncology clinics are addressing this gap through implementation of nutrition-focused quality improvement programs (QIPs). Weldishofer successfully implemented a QIP to evaluate feasibility of an evidence-based practice bundle involving nutrition assessment and counseling for high-risk patients receiving radiation therapy (Online Resource 1) [12]. A similar nutrition-focused QIP could be adapted to screen/intervene for patients receiving chemotherapy in outpatient clinics, particularly as clinic practices continue to evolve to integrate new therapies.

## Nutrition and the OCM

Consistently including quality nutrition care as part of the enhanced services OCM providers are paid to deliver could help close the nutrition gap in US ambulatory oncology settings and benefit patient and provider outcomes. CMS has acknowledged nutrition care is part of the OCM and that OCM requirements are intentionally high to allow areas like nutrition to be addressed as part of the model [13]. CMS has also identified referrals to dietitians as a resource some OCM providers are already employing [14] and that adding a nutritionist can develop the multidisciplinary team and augment staff to meet patient needs [15].

OCM oncology clinics must provide four types of enhanced services, use data to drive continuous quality improvement, and use certified electronic health record (EHR) technology (CEHRT) [16]. Detailed below are ways in which quality nutrition care specifically aligns with these OCM practice requirements and opportunities for improvement.

### Enhanced services

1. Patient navigation as a core function: this focuses on eliminating barriers to care; patients with cancer often develop multiple problems—including oral health conditions, gastrointestinal upsets, and metabolic changes—that can impact their nutrition and become barriers to tolerating treatment [17].
2. Care plans that include the Institute of Medicine (IOM) Care Management Plan’s 13 components: nutrition-related guidance aligns with many of these components (Online Resource 2).
3. 24/7 access to an appropriate clinician who can access the patient’s medical record: the patient’s nutrition care plan is part of the medical record but may not be readily visible. Access can be improved by assuring the nutrition care plan is in a structured format easily available in all views of the EHR.
4. Treatment therapies consistent with nationally recognized clinical guidelines: there are nutrition-specific oncology guidelines as well as nutrition recommendations in several site-specific oncology standards [18]. Yet many general US oncology treatment guidelines lack nutritional emphasis, suggesting nutrition is an under-utilized tool in cancer treatment.

### Use of data to drive continuous quality improvement and use of CEHRT

The OCM encourages and measures practices’ ability to identify and implement practice redesign strategies to improve the quality and experience of oncology care. Nutrition-focused QIPs can be useful in driving change as a part of practice redesign strategies, perfecting care processes in CEHRT, and advancing patient-centered care.

CMS uses specific quality measures to evaluate OCM practices on their quality of care and help determine performance-based payments. Nutrition can potentially impact each of the six quality measures for OCM providers in 2021 (Online Resource 3). Of note, unnecessary emergency department (ED) visits are a key quality metric and malnutrition is associated with more frequent and higher cost ED visits in cancer patients undergoing chemotherapy [19].

The OCM is still being tested and CMS uses mixed methods to evaluate the model’s performance, including administrative data and claims, patient surveys, and other inputs [20]. Patient surveys can include questions about whether the cancer therapy team provided additional services, such as help managing diet and exercise [14]. The Consumer Assessment of Healthcare Providers and Systems (CAHPS^R^) survey is required in the OCM and includes diet/energy specific questions [21].

## Conclusion

**Fig. 1:**
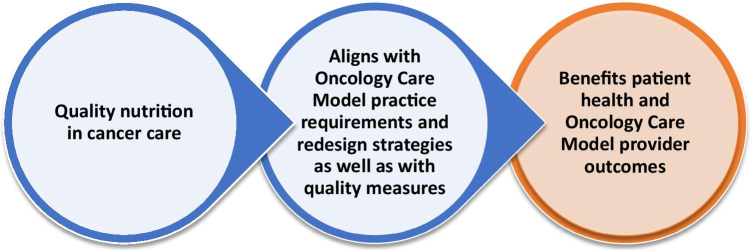
Quality nutrition care alignment with the Oncology Care Model

Malnutrition is common in patients with cancer and negatively impacts key health and quality of life outcomes. Yet lack of access to nutrition specialists in ambulatory oncology settings remains a critical issue for the US healthcare system. OCM providers can help address this by including nutrition in the enhanced services they deliver and incorporating quality nutrition care into their practice redesign activities (Fig. 1).

CMS is building on the OCM to launch the new Oncology Care First (OCF) payment model in 2021. As discussed, CMS has already acknowledged the role of the RDN and the potential impact of nutrition in the OCM which makes a powerful case for nutrition to be integral to the new OCF model. However, there is no need to wait, intentionally including nutrition can benefit patient health and provider outcomes now.

## Supplementary Information

Below is the link to the electronic supplementary material.

Supplementary file1 (PDF 122 KB)

## Data Availability

N/A

## Code availability

N/A
