# Supplementary material for: Commentary: Quality nutrition care is integral to the Oncology Care Model
Source: Support Care Cancer. 2021 Jul 23;29(12):7139–42. doi: 10.1007/s00520-021-06436-x (PMC8550270; doi:10.1007/s00520-021-06436-x)
Supplement: Supplementary file 1 — Supplementary file1 (PDF 122 KB) [file 520_2021_6436_MOESM1_ESM.pdf]

Title
Commentary: Quality Nutrition Care is Integral to the Oncology Care Model

Journal Name
Supportive Care in Cancer

Authors
Mary Beth Arensberg[1] (ORCID ID: 0000-0002-9825-2251)
Beth Besecker[1]
Laura Weldishofer[2]
Susan Drawert[3]

Author Affiliations
[1]Abbott Nutrition Division of Abbott, Columbus, Ohio, USA
[2]US Oncology, Cincinnati, Ohio, USA
[3]Abbott, Minneapolis, Minnesota, USA

Corresponding Author
Mary Beth Arensberg, mary.arensberg@abbott.com

Online Resource 1: Malnutrition assessment and intervention practices resulting from a nutrition-focused quality improvement program in a community radiation clinic [1]

**Assessment**
• All patients screened prior to initiation of radiation therapy
• Patients with radiation sites at high risk for malnutrition (head and neck, esophagus, lung with mediastinal lymph nodes in treatment field) identified
• At-risk patients receive baseline nutritional assessments and weekly re-assessments of nutritional intake, body mass, any nutrition-related side effects of treatment
• Weekly assessments of at-risk patients continue until their body mass stabilizes post treatment
**Intervention**
• At-risk patients receive counseling including personalized nutritional recommendations, based on guidelines from the Academy of Nutrition and Dietetics
• Nutrition recommendations include diet modifications (increased consumption of high protein, high calorie soft foods and oral nutrition supplements) as well as assessment criteria for potential gastrostomy tube placement and enteral feeding

1. Weldishofer L (2018) JL613. Nutrition assessment and management for head and neck cancer patients receiving definitive radiation therapy in the community setting. J Adv Pract Oncol 9:767-783.

Online Resource 2: Nutrition-related care guidance alignment with the 13 components in the Institute of Medicine (IOM) multidisciplinary patient Care Management Plan

**IOM Care Management Plan Component** [1]	**Nutrition-related Care Guidance**
1) Patient demographic information, medication list, and allergy list	Diet/nutrition information including supplement history, dietary recall
2) Diagnosis, including specific tissue information, relevant biomarkers, and staging information	Nutrition diagnosis including malnutrition, obesity, sarcopenia, sarcopenic obesity
3) Prognosis	Nutrition prognosis and potential for nutrition interventions to impact patient's nutrition status and diagnosis
4) Treatment goals (curative, life-prolonging, symptom control, palliative care)	Nutrition goals related to treatment goals, including active vs. palliative nutrition care
5) Initial plan for treatment and proposed duration, including specific chemotherapy drug names, doses, and schedules, as well as surgery and radiation therapy if applicable	Initial nutrition care plan and proposed duration, including recommendations related to oral nutrition supplements with dietary advice and/or other interventions to address malnutrition
6) Expected response to treatment	Expectation of nutrition interventions to improve malnutrition and/or other nutrition problems
7) Treatment benefits and harms, including common and rare toxicities, how to manage them, and short-term and late effects of treatment	Benefits of nutrition interventions, including strengthening ability to withstand treatment; recommendations on nutrition modifications/interventions that may help address treatment toxicities such as nausea, vomiting, and other side effects of treatment that could impact intake/nutrient absorption and increase risk for malnutrition
8) Quality-of-life information and patient's likely experience with treatment	Nutrition's potential to positively impact quality of life and help patient maintain energy, functionality, and ability to withstand treatments
9) Outline of who will take care of specific aspects of patient's care (cancer care team, geriatrics care team, other teams)	Outline of the role of the RDN in providing medical nutrition therapy and other team member responsibilities for nutrition care
10) Advanced care plans, including advanced directives and other legal documents	Advanced directives specific to enteral nutrition and total parenteral nutrition and withdrawal of nutrition support
11) Estimated total and out-of-pocket costs of cancer treatment	Estimated costs of nutrition interventions and explanation of potential economic benefits and cost savings associated with decreased risk for malnutrition and quality nutrition care
12) Psychosocial health plan, including psychological, vocational, disability, legal, or financial concerns	Impact of quality nutrition on psychological health and risk for disability
13) Detailed survivorship care plan, including a summary of treatment and information on recommended follow- up activities and surveillance, as well as risk reduction and health promotion activities	Nutrition guidance for survivorship, including recommended follow-up and guidance on reducing risk for malnutrition, improving nutritional status, and ultimately maintaining a healthy diet

1. Levit LA, Balogh EP, Nass SJ, Ganz PA (2013) Delivering High-Quality Cancer Care: Charting a New Course for a System in Crisis. National Academies Press, Washington, DC

Online Resource 3: Nutrition alignment with the Oncology Care Model (OCM) 2021 quality measures

**Quality measure name** (CMS, 2020)	**OCM measure #**	**Potential impact of nutrition**
Risk-adjusted proportion of patients with all-cause emergency department (ED) visits or observation stays that did not result in a hospital admission within the 6-month episode	OCM-2	Many common reasons for cancer patient ED visits are nutrition-related (such as nausea, vomiting, dehydration) [1]
Proportion of patients that died who were admitted to hospice for 3 days or more	OCM-3	Nutrition and hydration can be important for hospice patients, particularly if patients are malnourished [2]
Oncology: medical and radiation – pain intensity quantified (MIPS 133, NQF 0384)	OCM-4a	Emerging research identifies diet may be a regulator in chronic pain via management of inflammation/oxidative stress [3]
Oncology: medical and radiation – plan of care for pain (NQF 0383)	OCM-4b	Emerging research identifies diet may be a regulator in chronic pain via management of inflammation/oxidative stress [3]
Preventive care and screening: screening for depression and follow-up plan (CMS 2v8.1, NQF 0418)	OCM-5	There is a potential linkage between malnutrition and anxiety/depression underscoring the importance of malnutrition screening and intervention [4]
Patient-reported experience of care	OCM-6	Diet and energy questions are included in patient oncology surveys [5]

1. Bayrak E, Kitis Y (2018) The main reasons for emergency department visits in cancer patients. Med Bull Haseki, 56:6-13. https://doi.org/10.4274/haseki.83997
2. Bazzan A, Newberg AB, Cho WC, Monti, DA (2013) Diet and nutrition in cancer survivorship and palliative care. Evid Based Complement Alternat Med 2013:917647. https://doi.org/10.1155/2013/917647
3. Rondanelli M, Faliva MA, Miccono A, Naso M, Nichetti M, Riva A, Guerriero F, DeGeregori M, Peroni G, Perna S (2018) Food pyramid for subjects with chronic pain: foods and dietary constituents as anti-inflammatory and antioxidant agents. **Nutr. Res. Rev 31:1**31–151. https://doi.org/10.1017/S0954422417000270
4. Chabowski M, Polanski J, Jankowska-Polanska B, Janczak D, Rosirczuk J (2018) Is nutritional status associated with the level of anxiety, depression, and pain in patients with lung cancer? J Thorac Dis 10:2303-2310. https://doi.org/10.21037/jtd.2018.03.108
5. Agency for Healthcare Research and Quality (2020) CAHPS Cancer Care Survey. https://www.ahrq.gov/cahps/surveys-guidance/cancer/index.html. Accessed 1 March 2021.
